# Remarkable Case of Congenital Small-pox

**Published:** 1843-05

**Authors:** 


*Remarkable Case of Congenital Small-pox.*—A woman, twenty-four years of age, entered the Maternity Hospital in Paris to pass he first confinement. Labor commenced two days after her arrival; and after the lapse of fourteen hours (*jours*, says the original, but this is evidently an error) she was delivered of a female child. The face, scalp, and different parts of the child's body were covered by a pustular eruption, which was soon recognized to be veritable small-pox. The mother retained the marks of vaccination, and stated that she had never had the small-pox; nor during her pregnancy had she had connection with persons suffering under that disease, nor even heard of its prevalence in her neigborhood. Only, about eight or ten days before, she had gone to see a patient at La Pitié, near whom lay another patient in the small-pox. She had paid no attention to this circumstance till recalled to her recollection by minute inquiries. No untoward effects ensued, either to mother or child, and both left the hospital in perfect health soon afterwards—*Bulletin de l'Acad Royale.—Boston Med. Surg. Journ.*

